# Size Effect of Gold Nanoparticles in Catalytic Reduction of *p*-Nitrophenol with NaBH_4_

**DOI:** 10.3390/molecules181012609

**Published:** 2013-10-11

**Authors:** Chao Lin, Kai Tao, Dayin Hua, Zhen Ma, Shenghu Zhou

**Affiliations:** 1 Ningbo Institute of Materials Technology & Engineering, Chinese Academy of Sciences, Ningbo 315201, China; 2 Department of Physics, Faculty of Science, Ningbo University, Ningbo 315211, China; 3 Shanghai Key Laboratory of Atmospheric Particle Pollution and Prevention (LAP^3^), Department of Environmental Science and Engineering, Fudan University, Shanghai 200433, China

**Keywords:** gold catalysts, alumina, *p*-nitrophenol, nanoparticles, size effect

## Abstract

Gold nanoparticles (Au NPs) were prepared by reducing HAuCl_4_ with NaBH_4_. Their average particle sizes could be tuned in the range of 1.7 and 8.2 nm, by adjusting the amount of NaBH_4_ used during synthesis. The obtained Au NPs (colloids) were then loaded onto a commercial Al_2_O_3_ support to prepare Au/Al_2_O_3_ catalysts with tunable Au particle sizes. An optimal pH value (5.9) of the Au colloid solution was found to be essential for loading Au NPs onto Al_2_O_3_ while avoiding the growth of Au NPs. Au NPs and Au/Al_2_O_3_ catalysts were tested in the reduction of *p*-nitrophenol with NaBH_4_. Interestingly, the catalytic activity depended on the size of Au NPs, being the highest when the average size was 3.4 nm. Relevant characterization by UV-Vis, TEM, and XRD was conducted.

## 1. Introduction

Since Haruta and co-workers discovered that small gold nanoparticles (Au NPs) supported on some reducible oxides can be highly active catalysts for CO oxidation [1,2,3], heterogeneous catalysis by gold has attracted much attention [4,5,6]. Gold catalysts have found many applications in inorganic reactions (e.g., the water-gas shift reaction, ozone decomposition, selective oxidation of H_2_ to H_2_O_2_) and organic reactions (e.g., selective oxidation/reduction of organic compounds, carbon-carbon coupling) [5]. In particular, the application of gold catalysts in the synthesis of organic chemicals has been the subject of active research recently [7,8,9,10,11].

Supported gold catalysts were usually prepared by a deposition-precipitation method. Although the size of Au NPs can be kept small via that method, it is still difficult to control the size. Alternatively, Au NPs (colloids) with controllable sizes can be synthesized in a liquid phase, and then deposited onto a solid support [12,13,14,15]. That way, the influence of Au NP sizes on catalytic activity can be studied more conveniently.

In many cases, the catalytic activity increases as the average size of the Au NPs becomes smaller and smaller [16,17,18,19]. However, sometimes there exists an optimal particle size for a catalytic system. For instance, Valden *et al*. [20] dispersed Au NPs ranging from 1 to 6 nm in size on single-crystalline TiO_2_ surfaces, and found that the highest activity in CO oxidation was achieved when the Au particle size was between 2 and 4 nm. Laoufi *et al*. [21] investigated the catalytic activity of Au/TiO_2_ for CO oxidation, and found that the optimum Au particle size was 2.1 ± 0.3 nm.

Herein, Au NPs with tunable average particle sizes (1.7, 3.4, 5.7, 8.2 nm) were synthesized by reducing HAuCl_4_ with NaBH_4_, and then loaded onto a commercial Al_2_O_3_ support. The catalytic activities of unsupported Au NPs and Au/Al_2_O_3_ catalysts with different Au particle sizes were studied for the reduction of *p*-nitrophenol with NaBH_4_. An optimal Au particle size of 3.4 nm was identified.

## 2. Results and Discussion

### 2.1. Au NPs with Various Sizes

**Figure 1:**
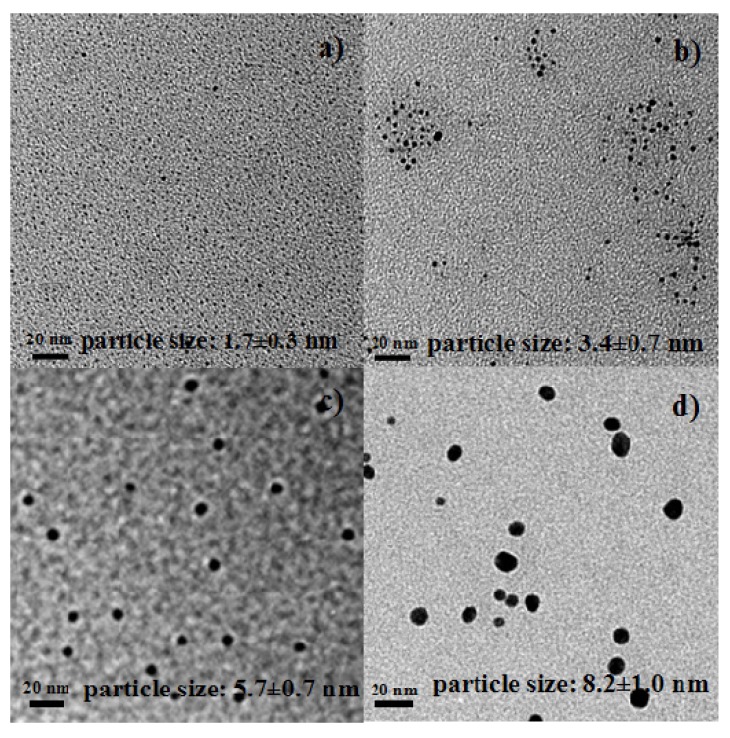
TEM images of Au NPs synthesized by mixing 0.06 mmol HAuCl_4_ with different amounts of NaBH_4_. (**a**) 0.4 mmol; (**b**) 0.5 mmol; (**c**) 1.0 mmol; and (**d**) 1.1 mmol NaBH_4_. Scale bars are 20 nm.

Au NPs (colloids) were prepared by reducing HAuCl_4_ (0.06 mmol) with NaBH_4_ (0.4, 0.5, 1.0, or 1.1 mmol). Figure 1 shows the TEM images of Au NPs synthesized with different amounts of NaBH_4_. The figure shows that the sizes of Au NPs can be tuned by adjusting the amount of NaBH_4_ added. For example, Au NPs with an average size of 1.7 nm were obtained when 0.06 mmol HAuCl_4_ and 0.4 mmol NaBH_4_ were mixed. On the other hand, the average size of Au NPs increased to 8.2 nm when 0.06 mmol HAuCl_4_ and 1.1 mmol NaBH_4_ were mixed. The average size and standard deviation were calculated based on 100 particles for each sample. The particle size distributions are shown in the Supplementary Materials. The size distribution of big Au NPs (8.2 ± 1.0 nm) is relatively wide. We attempted to synthesize the big Au NPs later, and also obtained a relatively wide size distribution (7.5 ± 1.8 nm, see Supplementary Materials).

**Figure 2:**
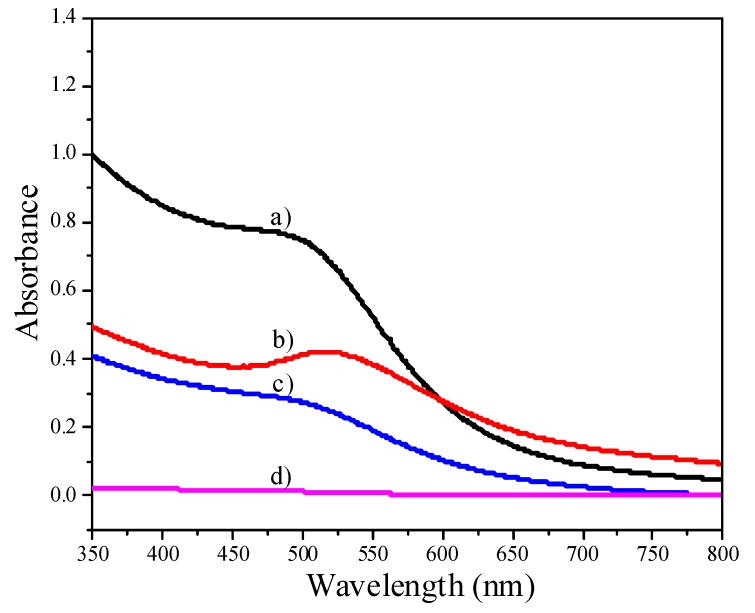
UV-Vis spectra of Au NPs. (a) Au NPs prepared with 0.4 mmol NaBH_4_; (b) Au NPs prepared with 1.0 mmol NaNH_4_; (c) the supernatant collected after adsorbing 5.7 nm Au NPs (at pH 8.2) onto Al_2_O_3_; (d) the supernatant collected after adsorbing 5.7 nm Au NPs (at pH 5.9) onto Al_2_O_3_.

The growth of gold nanoparticles as the amount of NaBH_4_ increases is also evident from UV-Vis data. When the amount of NaBH_4_ was 0.4 mmol, a shoulder band corresponding to small Au NPs appears, as shown in Figure 2a. The size of small Au NPs was estimated by TEM as 1.7 ± 0.3 nm (Figure 1a), and the size distribution was actually 1.1-2.4 nm (Supplementary Information). In the literature, Esumi *et al.* reported a similar shoulder band for 1.5 nm Au NPs [22]. In contrast, the Au NPs prepared with 1.0 mmol NaNH_4_ shows a distinctive absorption band centered at 525 nm (Figure 2b), corresponding to relatively bigger Au NPs [23].

### 2.2. Au/Al_2_O_3_ Catalysts

Au/Al_2_O_3_ catalysts were prepared by adsorbing Au NPs onto Al_2_O_3_ at room temperature. The pH value of the colloid solution has to be adjusted by aqueous HCl below the isoelectric point of Al_2_O_3_ (~7.5), to allow for the complete adsorption of Au NPs. We recorded UV-Vis spectra of the supernatant collected after adsorbing Au NPs (5.7 nm) onto the Al_2_O_3_ support. The supernatant still exhibited an absorption band centered at 516 nm when the pH value was 8.2 (Figure 2c), indicating the incomplete adsorption of Au NPs onto Al_2_O_3_. On the other hand, there was no absorption band corresponding to Au NPs in the supernatant when the pH value was 5.9 (Figure 2d), suggesting the complete loading of Au NPs onto Al_2_O_3_. This conclusion was also confirmed by ICP analysis.

**Figure 3:**
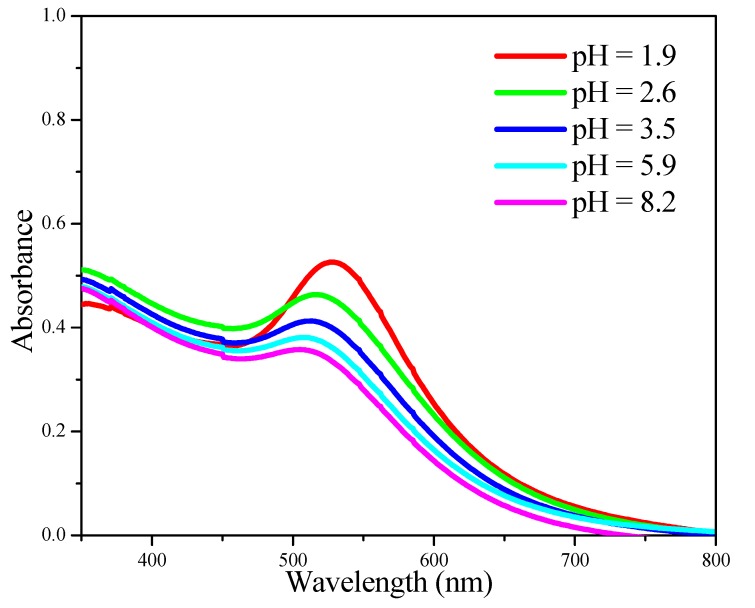
UV-Vis spectra recorded after adjusting the pH value of a 5.7 nm Au colloid solution by different amounts of aqueous HCl.

The effect of pH adjustment on the size of Au NPs was also investigated. As shown by the UV-Vis data in Figure 3, the absorption band corresponding to Au NPs became sharper and red-shifted as the pH value of the Au colloid solution decreased from 8.2 to 1.9, indicating the growth of Au NPs under very acidic conditions [24].

**Figure 4:**
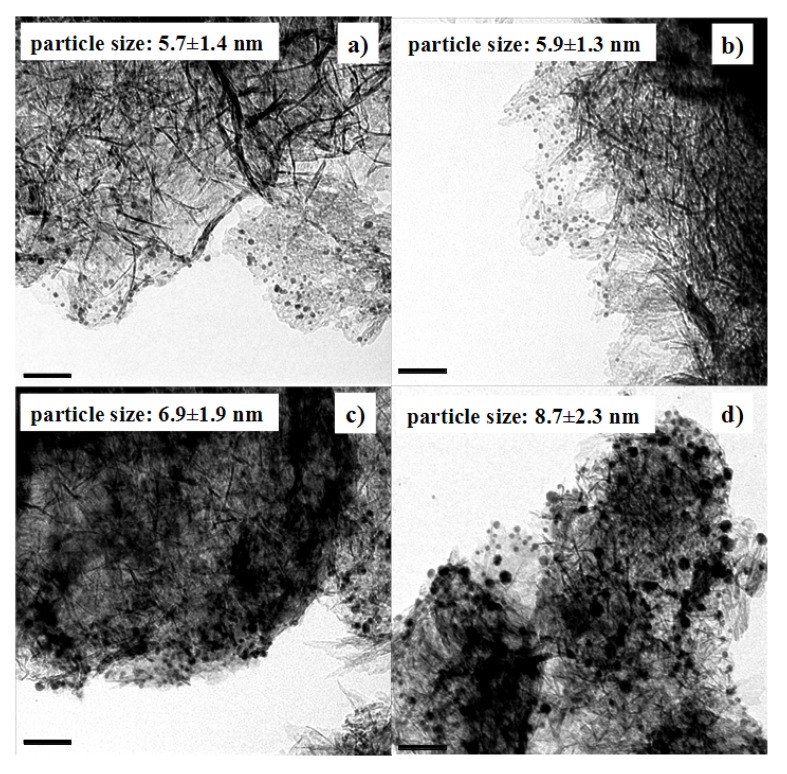
TEM images of Au/Al_2_O_3_ catalysts with Au colloid solutions adjusted to different pH values. (**a**) pH = 5.9; (**b**) pH = 3.5; (**c**) pH = 2.6; (**d**) pH = 1.9. The scale bars represent 50 nm. The size of the original Au colloid before pH adjustment is 5.7 nm.

The particle growth was further confirmed by TEM (Figure 4). The average particle size of Au NPs before the pH adjustment was 5.7 nm. The average particle size was still 5.7 nm when the pH value was adjusted to 5.9 and Au NPs were adsorbed onto Al_2_O_3_. In contrast, the average particle size became 8.7 nm when the pH value was 1.9. Therefore, pH 5.9 was chosen to load Au NPs onto Al_2_O_3_ in the following preparation. That way, we can load Au NPs onto Al_2_O_3_ completely, while keeping their original size.

**Figure 5:**
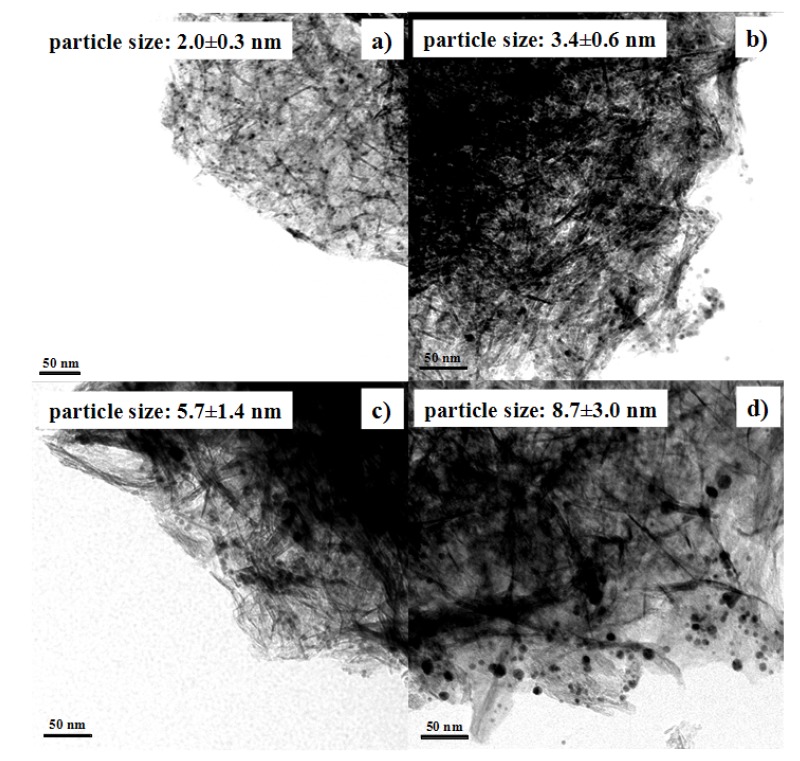
TEM images showing Au/Al_2_O_3_ catalysts prepared from Au NPs with various particle sizes: (**a**) 1.7 nm; (**b**) 3.4 nm; (**c**) 5.7 nm; (**d**) 8.2 nm.

Au NPs with different average sizes were used to prepare Au/Al_2_O_3_ catalysts. The pH value of Au colloid solutions was adjusted to 5.9 in the preparation. As shown in Figure 5, the average sizes of gold nanoparticles supported onto Al_2_O_3_ were 2.0, 3.4, 5.7, and 8.7 nm, respectively. Therefore, the Au particle sizes were basically maintained after loading Au NPs onto the Al_2_O_3_ support.

**Figure 6:**
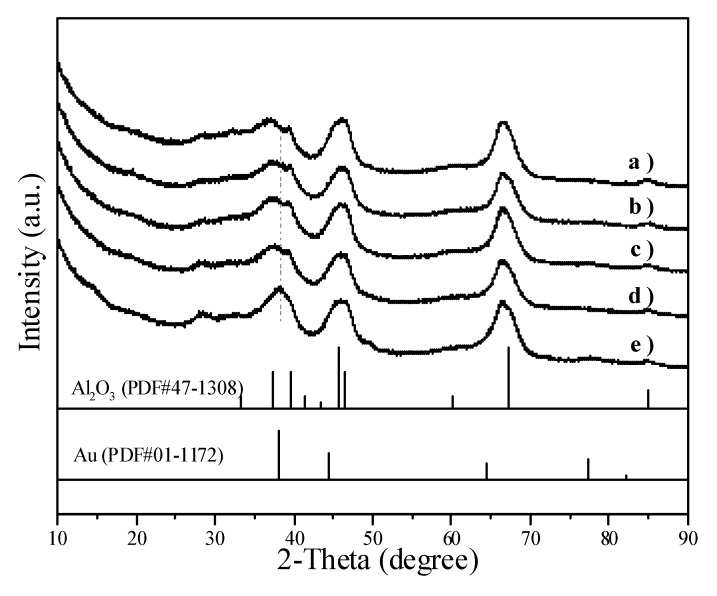
XRD patterns showing (a) Al_2_O_3_ and Au/Al_2_O_3_ catalysts with different Au particle sizes: (b) 2.0 nm; (c) 3.4 nm; (d) 5.7 nm; (e) 8.7 nm.

Figure 6 illustrates the XRD patterns of Au/Al_2_O_3_ catalysts. The peaks corresponding to metallic Au overlapped with the peaks of Al_2_O_3_. When the average Au particle size increased from 2.0 nm in Figure 6b to 8.7 nm in Figure 6e, the Au peak at 2θ = 38.26° became visible.

### 2.3. Catalytic Reduction of p-Nitrophenol

**Figure 7:**
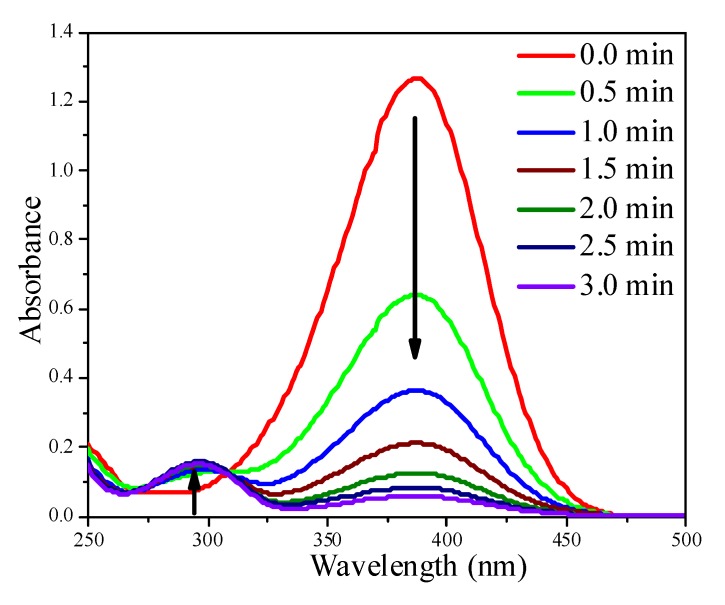
The time course of UV-Vis spectra of the 4-NP reduction system using 1.0 mL 5.7 nm Au NP colloid solution containing 0.10 mg Au NPs.

The reduction of *p*-nitrophenol (4-NP) with NaBH_4_ was used to evaluate the catalytic activity of Au colloids and supported Au/Al_2_O_3_ catalysts. The reduction process was monitored by UV-Vis. In the UV-Vis spectra, the absorption at 384 nm corresponds to 4-NP (the reactant), whereas the absorption at 296 nm corresponds to *p*-aminophenol (4-AP), the product. No reaction happened in the absence of catalyst (data not shown). Figure 7 illustrate the absorbance changes at 384 and 296 nm in the presence of Au NPs (colloids). The Au NPs were active, evident from the faster drop of the absorbance at 384 nm. Since the amount of NaBH_4_ was 100 times higher than that required by the stoichiometry, the reaction was pseudo first-order to 4-NP.

*Reaction conditions*: 4-NP: 0.150 mmol; NaBH_4_: 15.0 mmol; Au: 0.10 mg; total volum of aqueous solution: 100 mL; T: 25 °C.

Table 1 summarizes the turnover frequency (TOF) of Au NPs with different Au particle sizes. The TOF was defined as in [25,26]. For TOF calculation, the reaction time was 60 seconds. In an earlier report, TOF increased as the particle size decreased [25]. In our current study, the TOF value was the highest when the particle size was 3.4 nm.

**Table 1:** Gold particle size and the corresponding TOF for the 4-NP Reduction.

Particle size (nm)	Reaction time (s)	Conversion (%)	TOF (moles·g^−1^·s^−1^)
1.7	60	73.0	1.83 × 10^−2^
3.4	60	82.5	2.06 × 10^−2^
5.7	60	71.3	1.78 × 10^−2^
8.2	60	62.3	1.56 × 10^−2^

**Figure 8:**
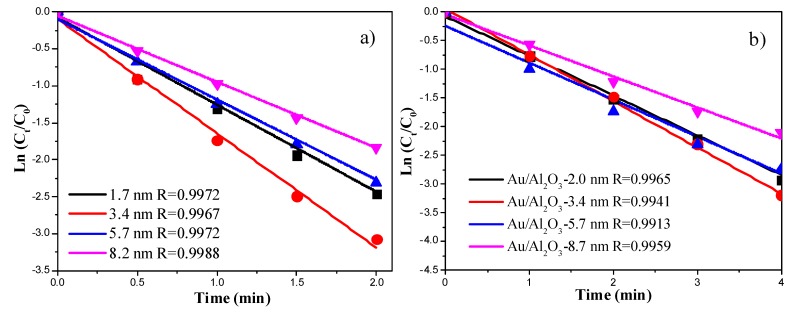
(**a**) Plots of the Ln (C_t_/C_0_) versus reaction time for Au NPs with different sizes; (**b**) Plots of the Ln (C_t_/C_0_) *versus* reaction time for Au/Al_2_O_3_ with different Au particle sizes.

**Figure 9:**
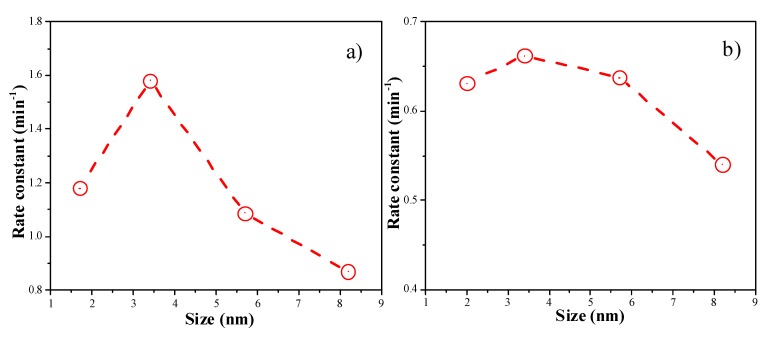
(**a**) the correlation between the rate constants and the Au particle sizes of Au colloids; (**b**) the correlation between the rate constants and the Au particle sizes of Au/Al_2_O_3_ catalysts.

Figure 8 shows the plots of the Ln (C_t_/C_0_) versus reaction time of 4-NP reduction reaction catalyzed by Au colloids and Au/Al_2_O_3_. The linear coefficients were all above 0.99, suggesting that the catalytic reduction of 4-NP follows pseudo first-order kinetics. Figure 9 demonstrates the correlation between the rate constant (obtained from the slopes in Figure 8) and the particle size of Au nanocatalysts. The highest rate constants were observed when the Au particle size was around 3.4 nm. The presence of an optimum Au particle size was also observed in Au/TiO_2_ catalysts for low temperature CO oxidation [20,21]. The reason why Au NPs smaller than 2 nm were less active is probably due to the deviation of metallic nature of such small Au NPs [20,21]. The reduction of *p*-nitrophenol requires metallic gold nanoparticles. Alternatively, this could be due to the different structures of the Au NPs as their sizes decrease. This aspect can be studied in more details in the future.

## 3. Experimental

### 3.1. Chemicals

All chemicals were used as received. HAuCl_4_·4H_2_O (99.999%), NaBH_4_ (AR), and *p*-nitrophenol (AR) were purchased from Aladdin Co (Shanghai, China). Polyvinylpyrrolidone (PVP, MW-58000) and aqueous HCl was purchased from Sinopharm Chemical Reagent Co (Shanghai, China). Al_2_O_3_ (surface area 350 m^2^/g, pore volume 0.8 ml/g) was purchased from Shandong Zibo Chemical Co (Shandong, China).

### 3.2. Catalyst Preparation

#### 3.2.1. Preparation of Au NPs

Au NPs with different sizes were prepared by a reported method [27]. HAuCl_4_·4H_2_O (0.06 mmol) and PVP (10.0 mg) were dissolved in deionized water (95.0 g) in a round-bottom flask, followed by stirring for 30 min. Aqueous NaBH_4_ (5 mL) containing 1.0 mmol NaBH_4_ was then injected. The color of solution turned to dark red instantly. The solution was further stirred for 1 h to obtain 5.7 nm Au NPs. Au NPs with average sizes of 1.7, 3.4, and 8.2 nm were obtained by adding 0.4, 0.5, and 1.1 mmol NaBH_4_, respectively.

#### 3.2.2. Preparation of 1.0 wt% Au/Al_2_O_3_ Catalysts

Typically, Au NPs (30.0 g) containing 0.018 mmol Au were transferred into a 3-neck round-bottom flask, and then a certain amount of HCl (10%) was added to adjust the pH value of the colloid solution. After that, Al_2_O_3_ (0.355 g) was added. The slurry was stirred for 1 h. Au/Al_2_O_3_ catalysts were collected by repeated centrifugation and washing.

### 3.3. Characterization

XRD patterns were collected on a Bruker AXS D8 Advance diffractometer using Cu Kα radiation. TEM and HRTEM images were obtained by a JEOL 2100 transmission electron microscope operated at 200 kV. Before imaging, the samples were dispersed in ethanol by sonication, and a few drops of the dispersion were dropped onto a carbon-coated Cu grid followed by solvent evaporation in air at room temperature. UV-Vis absorption spectra were recorded on a UV-3300 spectrophotometer.

### 3.4. Catalytic Reduction of p-Nitrophenol with NaBH_4_

Typically, aqueous *p-*nitrophenol (50.0 mL, 0.15 mmol *p*-nitrophenol) and aqueous NaBH_4_ (50.0 mL, 15.0 mmol NaBH_4_) were transferred into a 3-neck round-bottom flask. After stirring for several minutes, Au colloids (1.0 mL, 0.10 mg Au NPs) or 1.0 wt% Au/Al_2_O_3_ (10.0 mg) was transferred into the flask. The mixture was continuously stirred. Solution (1.0 mL) was sampled at certain intervals, diluted immediately with cold deionized water (9.0 mL, below 5 °C), and measured immediately by an ultraviolet spectrophotometer in the range of 250–500 nm. The absorption of *p*-nitrophenol was observed at 384 nm, and the absorption of the product *p*-aminophenol was observed at 296 nm.

## 4. Conclusions

Au NPs ranging in size from 1.7 to 8.2 nm were synthesized by reducing HAuCl_4_ with different amounts of NaNH_4_. The pH value of the colloid solutions was adjusted to 5.9 in order to allow for complete adsorption of Au NPs onto Al_2_O_3_ support while keeping the Au particle size intact. Au NPs and Au/Al_2_O_3_ catalysts with an average Au particle size of 3.4 nm showed the highest activities in reduction of 4-nitrophenol by NaBH_4_.

## Supplementary Materials

Supplementary materials can be accessed at: http://www.mdpi.com/1420-3049/18/10/12609/s1.
