# Supplementary material for: Size Effect of Gold Nanoparticles in Catalytic Reduction of p-Nitrophenol with NaBH4
Source: Molecules. 2013 Oct 11;18(10):12609–20. doi: 10.3390/molecules181012609 (PMC6269978; doi:10.3390/molecules181012609)

Supplementary Materials

Figure S1. TEM image and particle size distribution of Au NPs (1.7 ± 0.3 nm).

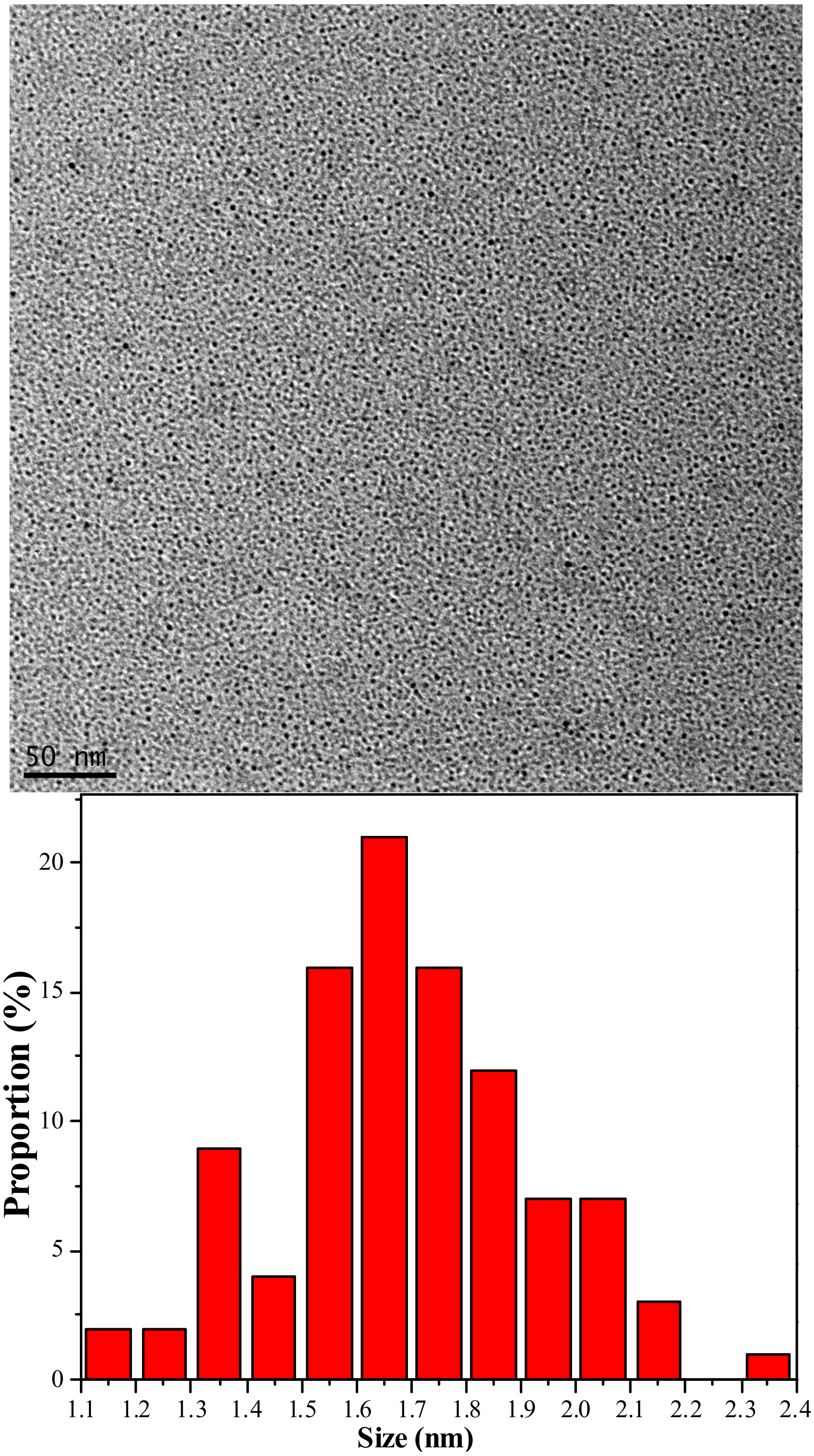

Figure S2. TEM image and particle size distribution of Au NPs (3.4 ± 0.7 nm).

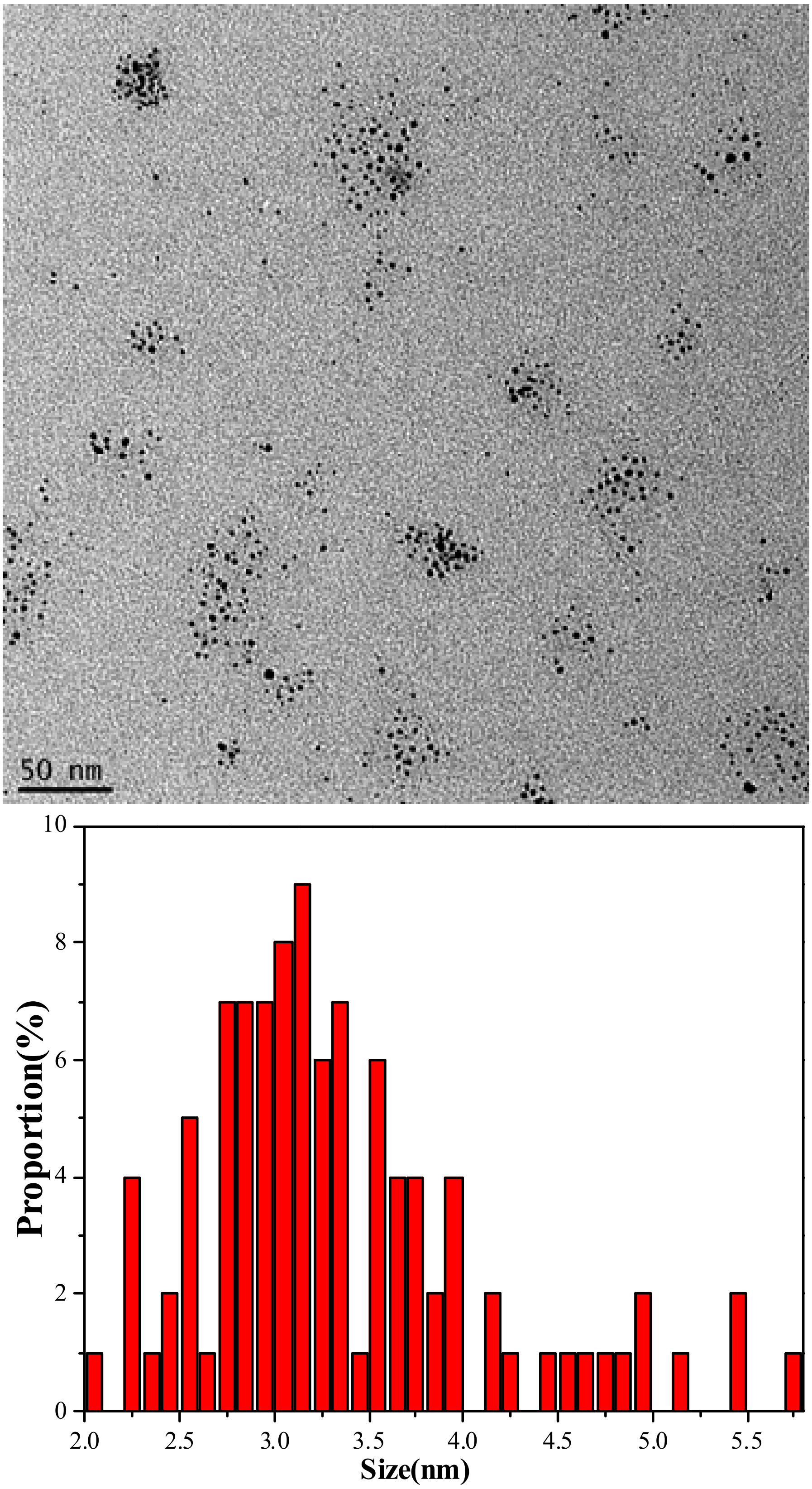

Figure S3. TEM image and particle size distribution of Au NPs (5.7 ± 0.7 nm).

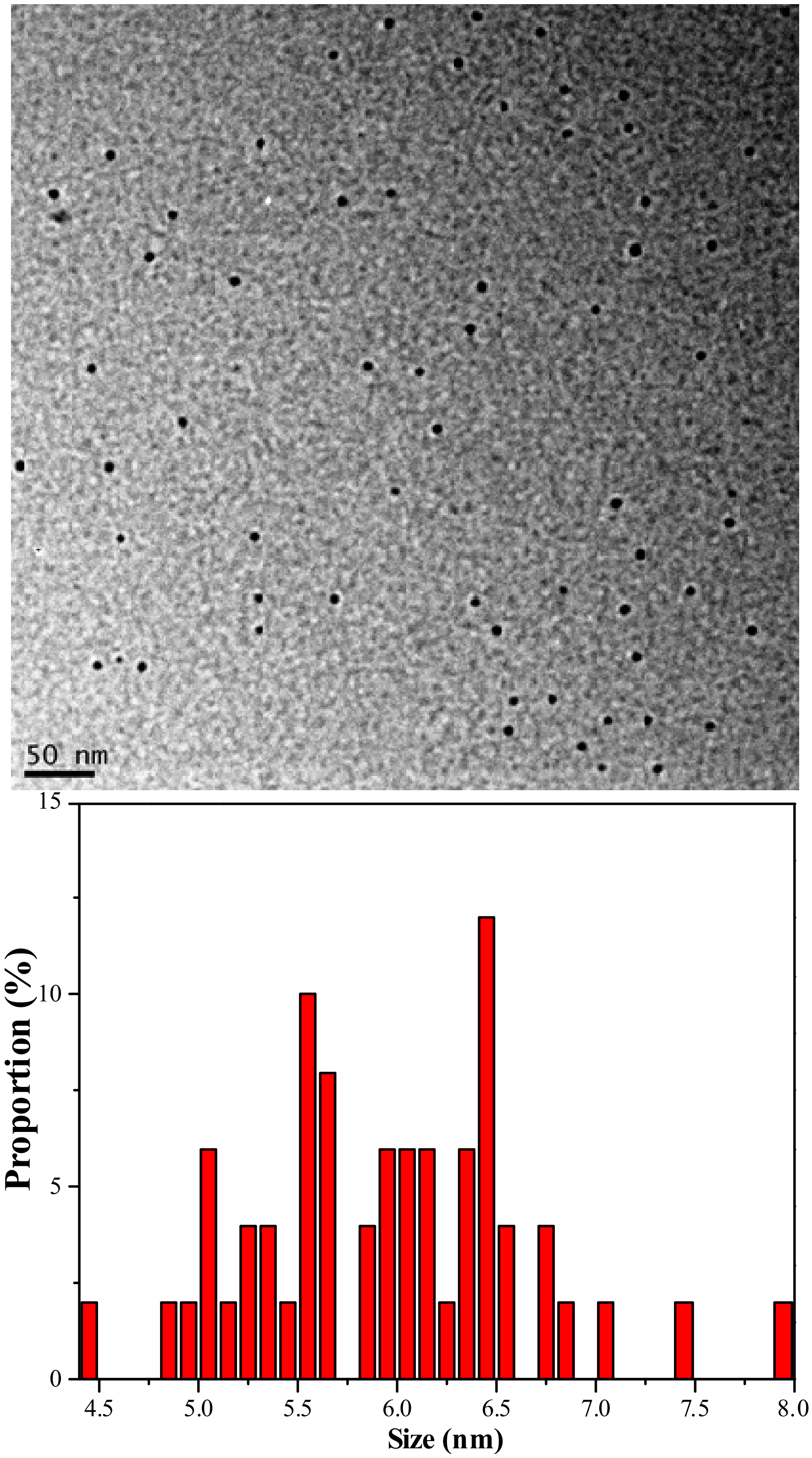

Figure S4. TEM image and particle size distribution of Au NPs (batch #1: 8.2 ± 1.0 nm).

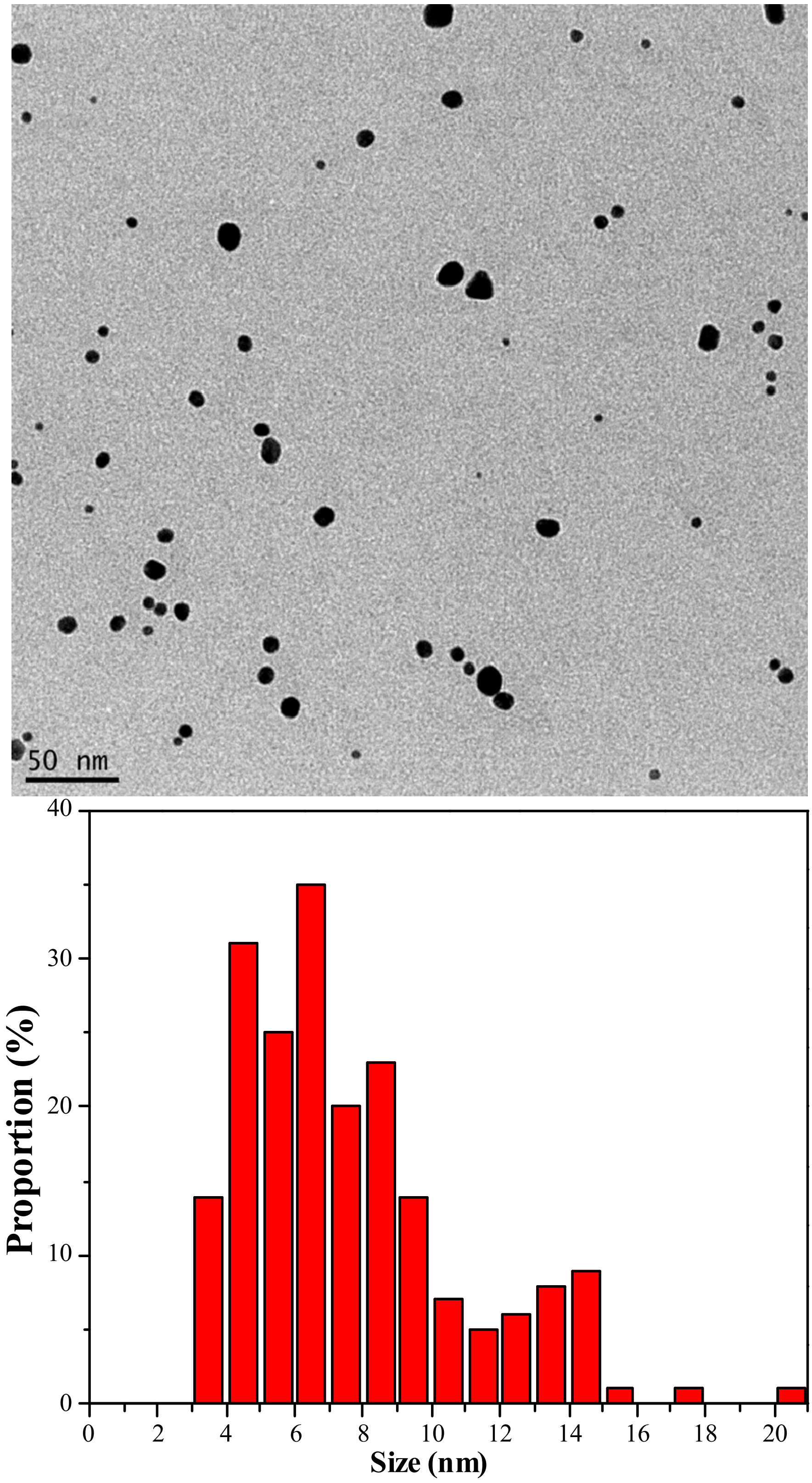

Figure S5. TEM image and particle size distribution of Au NPs (batch #2: 7.5 ± 1.8 nm).

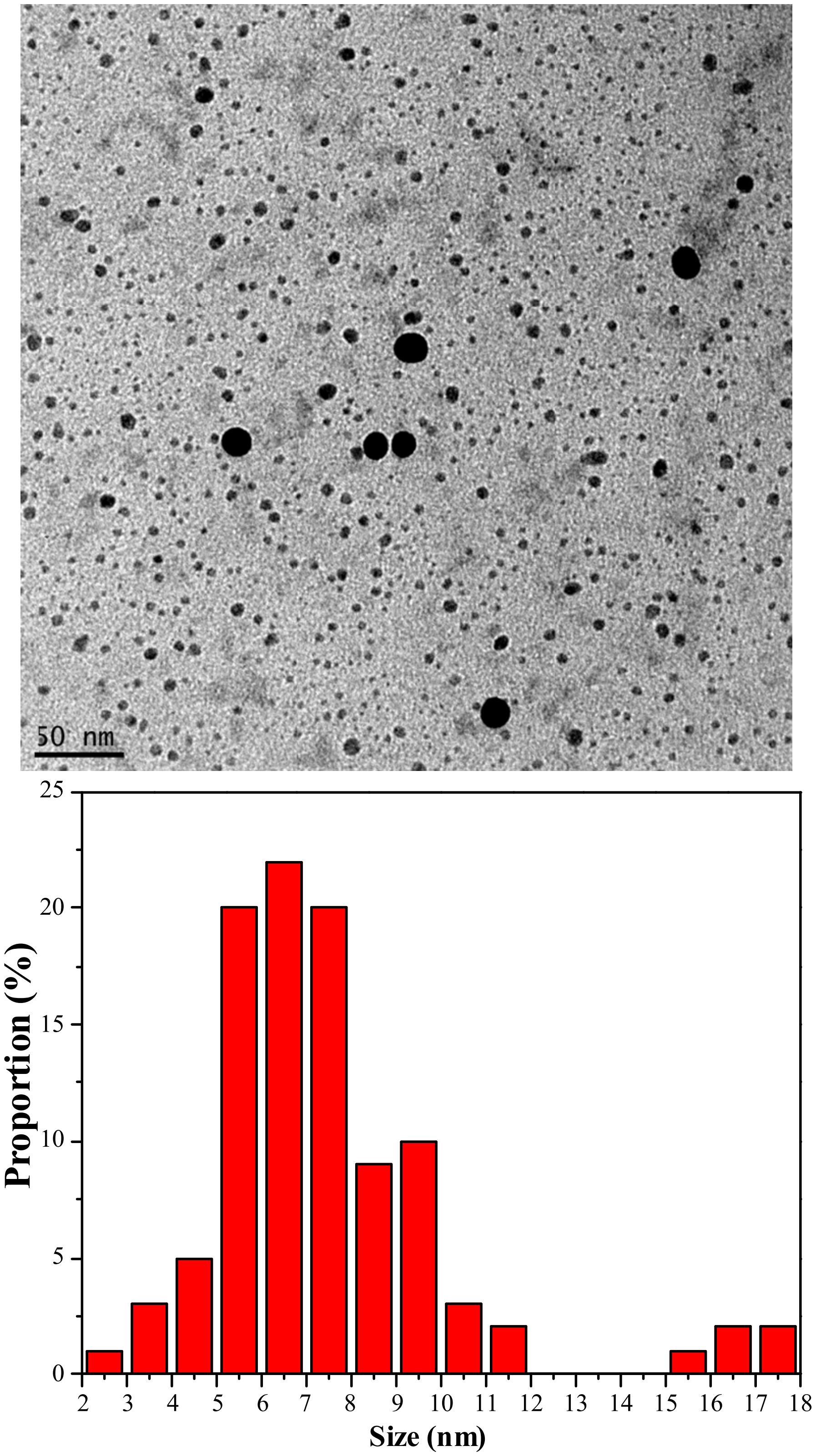

Figure S6. TEM image processing: from the raw images to the ones shown in Figure 1.

(**a**) **AuNPs——1.**7 ± 0.3 nm

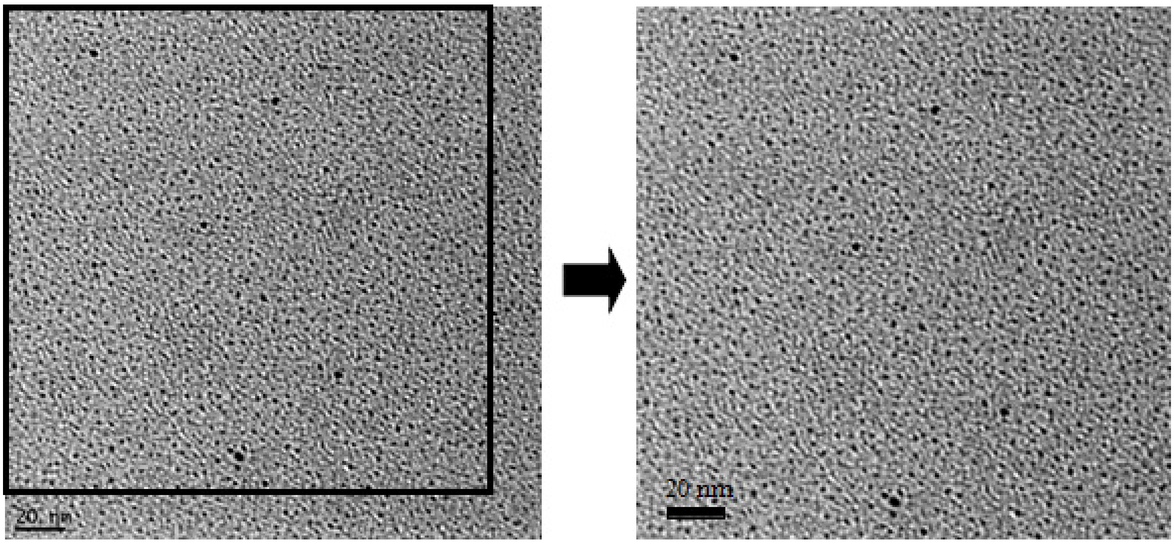

(**b**) **AuNPs——3.**4 ± 0.7 nm

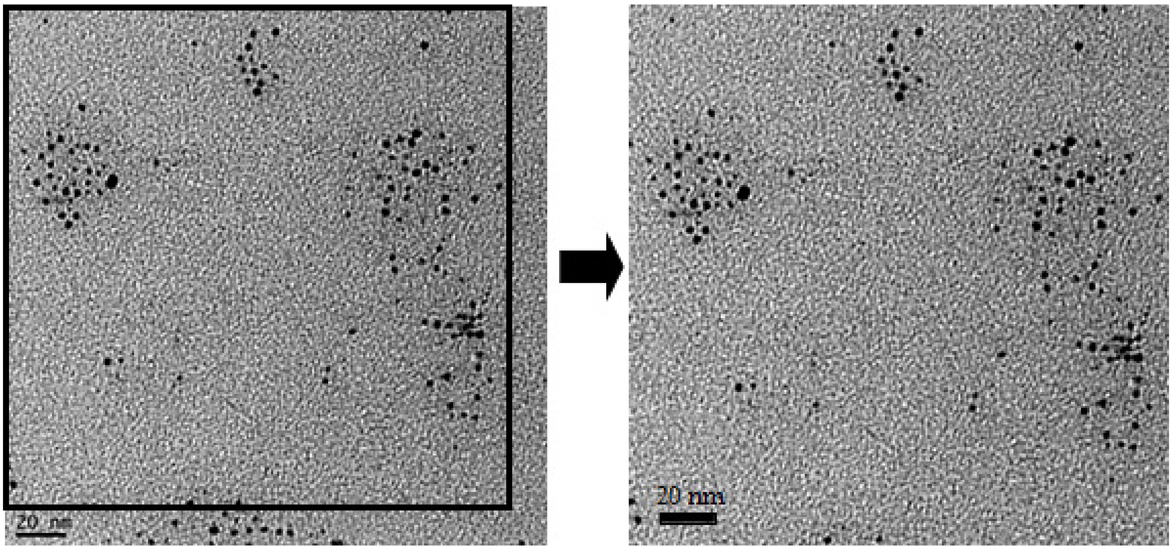

(**c**) **AuNPs——5.**7 ± 0.7 nm

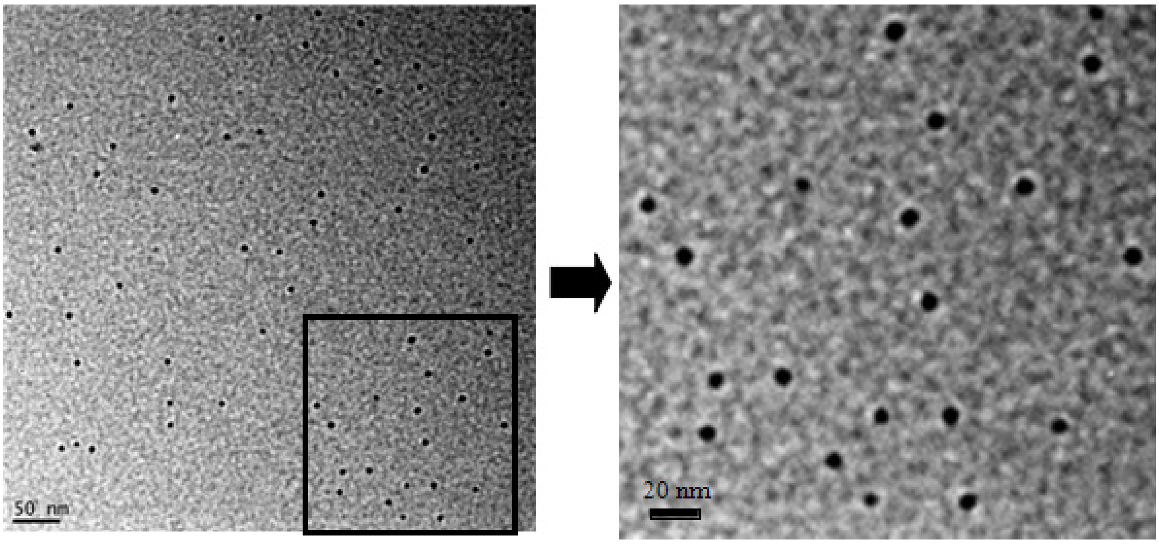

Figure S6. *Cont.*

(**d**) AuNPs (batch #1)——8.2 ± 1.0 nm

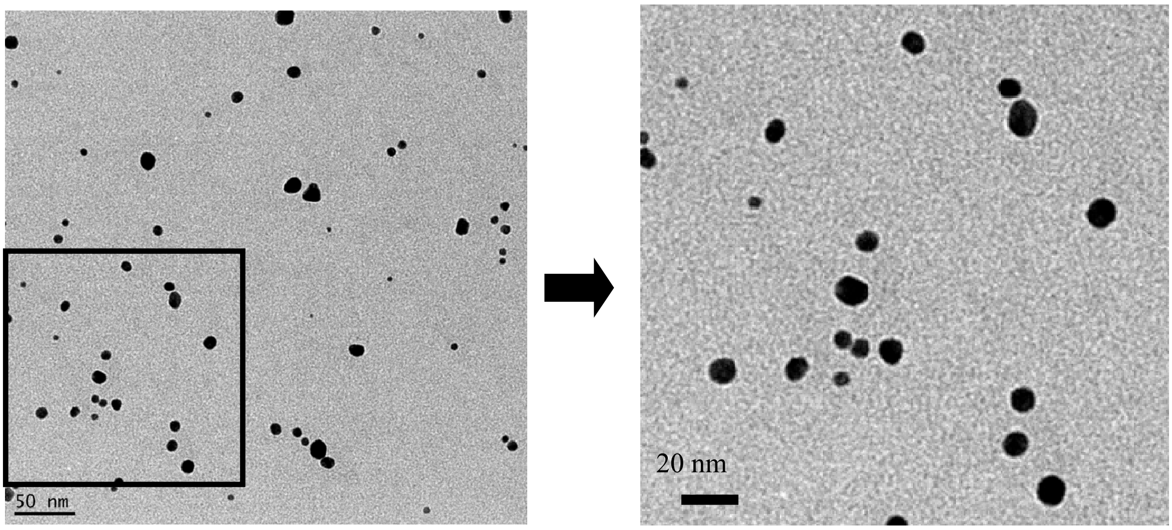

Supplement: Supplementary file 1 [file molecules-18-12609-s001.pdf]
